# Bioprotective Lactic Acid Bacteria and Lactic Acid as a Sustainable Strategy to Combat *Escherichia coli* O157:H7 in Meat

**DOI:** 10.3390/foods12020231

**Published:** 2023-01-04

**Authors:** Ayelen A. Baillo, Lucia Cisneros, Julio Villena, Graciela Vignolo, Silvina Fadda

**Affiliations:** 1 Laboratory of Technology of Meat and Meat Products, Centro de Referencia para Lactobacilos (CERELA-CONICET), San Miguel de Tucuman T4000ILC, Argentina; 2 Laboratory of Immunobiotechnology, Centro de Referencia para Lactobacilos (CERELA-CONICET), San Miguel de Tucuman T4000ILC, Argentina

**Keywords:** lactic acid bacteria, enterohemorrhagic *Escherichia coli*, bioprotection, meat, lactic acid

## Abstract

Human infection by Enterohemorrhagic *Escherichia coli* (EHEC) constitutes a serious threat to public health and a major concern for the meat industry. Presently, consumers require safer/healthier foods with minimal chemical additives, highlighting the need for sustainable solutions to limit and prevent risks. This work evaluated the ability of two antagonistic lactic acid bacteria (LAB) strains, *Lactiplantibacillus plantarum* CRL681 and *Enterococcus mundtii* CRL35, and their combination in order to inhibit EHEC in beef (ground and vacuum sealed meat discs) at 8 °C during 72 h. The effect of lower lactic acid (LA) concentrations was evaluated. Meat color was studied along with how LAB strains interfere with the adhesion of *Escherichia coli* to meat. The results indicated a bacteriostatic effect on EHEC cells when mixed LAB strains were inoculated. However, a bactericidal action due to a synergism between 0.6% LA and LAB occurred, producing undetectable pathogenic cells at 72 h. Color parameters (a*, b* and L*) did not vary in bioprotected meat discs, but they were significantly modified in ground meat after 24 h. In addition, LAB strains hindered EHEC adhesion to meat. The use of both LAB strains plus 0.6% LA, represents a novel, effective and ecofriendly strategy to inactivate EHEC in meat.

## 1. Introduction

Human infection with enterohemorrhagic *Escherichia coli* (EHEC) is caused by the ingestion of contaminated food, such as milk, vegetable products and, particularly, meats [1]. Due to the low infective dose (10–100 CFU), it represents a serious problem for public health and a major concern for the sustainability of the meat industry [2]. This pathogen is found naturally, colonizing the gastrointestinal tract of different ruminants [3], and is associated worldwide with outbreaks, causing from mild diarrhea to hemorrhagic colitis, thrombocytopenia and hemolytic uremic syndrome (HUS) [4,5]. In several regions of the world, including regions in South American countries, EHEC O157:H7 remains the main serotype associated with human infections, with a significant number of HUS cases [6]. In Argentina, out of 290 cases of HUS reported in 2019, 232 (80%) corresponded to children under 5 years of age, reaching an incidence of 6.23 cases per 100,000 children [7]. Although this number is lower than the average of the last 5 years, it still is the most acute renal failure causative in pediatrics and the second cause of renal transplantation in children [8].

Potential vehicles for EHEC are raw or undercooked meat, mainly ground meat, contaminated during processing [9]. Bacterial attachment to solid surfaces is a complex process, usually involving more than one mechanism influenced by many factors, including bacterial surface properties, solid surface characteristics and the environment as well as the presence of other bacteria [10,11,12]. A two-step model is generally used to explain the attachment of bacterial cells to a surface: reversible nonspecific attachment, which allows easy removal of bacterial cells by rinsing and turbulent fluid flow, followed by irreversible specific attachment, which requires greater forces to remove bacterial cells. These forces can be physical (scraping/scrubbing) or chemical using cleaners and disinfectants, which can be aggressive on the food [10,12,13].

The food industry uses different methods to control and/or inhibit pathogenic or spoilage microorganisms in order to extend shelf life and provide safe food for consumption. Most applied interventions consist in temperature manipulation and implementation of chemical treatments such as the use of organic acids and basic salts [14]. Discoloration of meat remains an important issue, particularly when high concentrations of organic acids are used for bacterial decontamination. Color stability is a matter of concern in the meat industry as it is the main quality attribute that affects the buying decision of consumers [15].

Nowadays, consumer demand for safer foods, with minimal processing and fewer chemical additives but with an undamaged sensorial quality, constitutes a great challenge [16]. In this context, new techniques, such as biopreservation, have gained increasing attention due to the possibility of providing a sustainable and ecological solution. Bioprotective strategies propose the use of antagonistic microorganisms or their metabolic products to control undesirable organisms (pathogens and contaminants) in order to prolong shelf life and improve food safety, without altering the sensory characteristics of the final product [17]. In this regard, some strains of lactic acid bacteria (LAB), known as bioprotective LAB, are used to control and/or inhibit detrimental microorganisms [18,19,20]. Moreover, they are recognized as safe when added to food and should exert its antimicrobial action without affecting quality and sensory characteristics of the food [20,21,22].

LAB are known to produce a wide array of antimicrobial substances, such as organic acids, ethanol, diacetyl, hydrogen peroxide, reuterin, reutericyclin, antifungal compounds and bacteriocins [23]. However, not only are these metabolic products responsible for the control and/or inhibition of undesirable microorganisms, the presence of LAB interacting with major pathogens such as *Escherichia coli* and *Listeria monocytogenes* is able to displace them as previously demonstrated [24,25]. In fact, the antagonistic ability of *Lactiplantibacillus* (*L.*) *plantarum* CRL681 and *Enterococcus* (*E.*) *mundtii* CRL35 against *Escherichia coli* O157:H7 and *Listeria monocytogenes* was reported in meat-models [2,25,26].

The present study was undertaken to determine the effectiveness of *L. plantarum* CRL681 and *E. mundtii* CRL35 and their combination on the inactivation of *Escherichia coli* O157:H7 in fresh meat (minced beef and vacuum-packed beef discs) stored at 8 °C during 72 h. The effect of lactic acid was also evaluated. Meat color was studied along with how the presence of LAB strains interferes with the adhesion of *Escherichia coli* cells to meat.

## 2. Materials and Methods

### 2.1. Bacterial Strains and Culture Conditions

*Lactiplantibacillus plantarum* CRL681 and *Enterococcus mundtii* CRL35 were isolated from Argentine fermented sausages and artisanal cheese from Northwest Argentina, respectively [27,28]. Both strains belong to the culture collection of CERELA-CONICET and were used as bioprotective cultures in this work. The CRL681 and CRL35 strains were selected for their technological properties [29] and their ability to inhibit *Escherichia coli* O157: H7 and *Listeria monocytogenes* [2]. The cultures were obtained from the stock stored at −80 °C by transferring three times in MRS broth (Merck, Buenos Aires, Argentina) and incubating at 30 °C for 24 h; the last subculture was incubated overnight (O/N). 

*Escherichia coli* O157:H7 NCTC12900 (National Type Culture Collection, Colindale, London, UK) was selected as a pathogen model; it is unable to produce enterotoxins Stx1 or Stx2 [30]. The culture was obtained by making three transfers in Luria Bertani (LB) medium and incubating at 37 °C with agitation for 24 h; the last subculture was incubated O/N and was used for the in situ assays.

### 2.2. Experimental Systems: Ground Meat and Meat Discs

Experimental systems were designed to analyze the inhibitory effect of bioprotective LAB strains and lactic acid (0.6%) as an adjuvant against EHEC using a storage temperature of 8 °C to approximate real meat processing conditions. 

**Figure 1:**
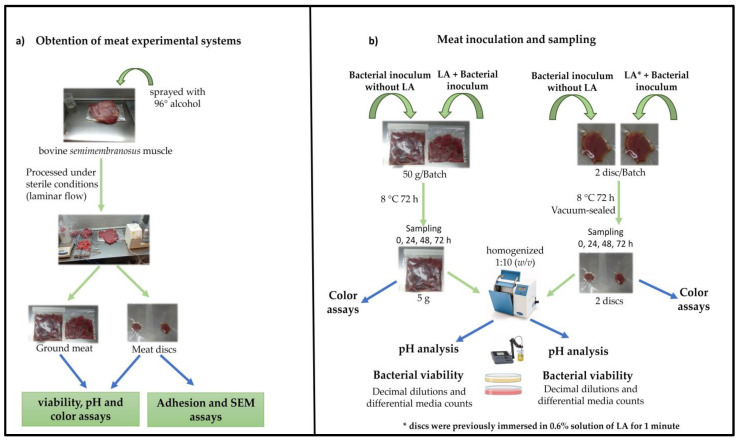
Schematic description of (**a**) experimental systems preparation and (**b**) meat inoculation and analysis.

For this purpose, bovine *semimembranosus* muscle was obtained from a local abattoir. After removing fat and connective tissue in aseptic conditions, it was processed to prepare the experimental systems. Briefly, the surface of the meat pieces as well as the working tools (knives, forceps) were sprayed with 96° alcohol and dried in laminar flow and subjected to UV radiation for 30 min. The top layer of meat (approximately 2 cm) was discarded using a knife (regularly flamed). The remaining meat was aseptically divided in two portions: the first one was cut into small pieces and processed to obtain ground meat (GM); the other portion was sliced into approximately 0.5 cm wide steaks to obtain meat discs by using a stainless steel circle tool (3 cm diameter, 1 cm thick). The ground meat and meat discs were placed separately in bags and sealed at a final vacuum of 99% using a Turbovac 320 ST vacuum packaging machine (Howden Food Equipment, Holland) and stored at −20 °C until required for the experiments (Figure 1). Control of microbial loads of the meat systems was carried out by plating on plate count agar (PCA) (Merck, Buenos Aires, Argentina).

### 2.3. Meat Inoculation and Sampling

#### 2.3.1. Ground Meat Assays

Portions of 50 g of GM were aseptically weighted for each batch. Each bag containing GM was added with 5 mL of physiological solution inoculated with each bacterium (approximately 1 × 10^6^ CFU/g for LAB and 1 × 10^4^ CFU/g for EHEC). Additionally, 5 mL of physiological solution was added with lactic acid (LA) to reach a final concentration of 0.6% in the GM. Controls inoculated without lactic acid addition and controls containing neither bacteria nor lactic acid were prepared.

The bags containing inoculated GM samples were manually mixed. The batches were incubated for 72 h at 8 °C and samples were taken at 0, 24, 48 and 72 h. The resulting 14 batches are detailed in Table 1. For each sampling time, 5 g of GM was homogenized 1:10 (*w*/*v*) with sterile distilled water using a laboratory blender (Stomacher 400, London, UK) for 2 min. Bacterial enumeration and pH measurements were determined in meat slurries. Figure 1 describes the steps of this process.

#### 2.3.2. Disc Meat Assays

Meat discs (each side) were inoculated with 50 µL of a cell suspension containing LAB (1 × 10^6^ CFU/cm^2^) and EHEC (1 × 10^4^ CFU/cm^2^) and dispersed with a Drigalski spatula. Un-inoculated controls were added with physiological solution (50 µL). The discs tested with the addition of 0.6% lactic acid (LA) were previously immersed in the LA solution and allowed to dry for a few minutes in the laminar flow. The resulting 14 batches are specified in Table 1. Finally, the packed discs were sealed at a final vacuum of 99% using a Turbovac 320 ST vacuum-packaging machine and incubated for 72 h at 8 °C. Samples were taken at 0, 24, 48 and 72 h. Each sample contained two meat discs per batch/time. Each meat disc was processed individually by homogenization 1:10 (*w*/*v*) with sterile distilled water using a laboratory blender (Stomacher 400, London, UK) for 2 min. This meat homogenate was used for bacterial enumeration and pH measurements (Figure 1).

### 2.4. Bacterial Counts and pH Measurement

For bacterial enumeration, decimal dilutions were prepared and plated on MRS agar (Merck, Buenos Aires, Argentina) for LAB and MacConkey with sorbitol (Merck, Buenos Aires, Argentina) for EHEC, then incubated for 48 h at 30 °C and 37 °C, respectively. The pH values of meat experimental systems were measured by using a Metrohn 692 pH/Ion Meter. Bacterial viability was expressed as log CFU/g for ground meat and log CFU/cm^2^ for meat discs.

### 2.5. Color

In order to determine the effect of the addition of LAB with and without lactic acid on the meat systems, colorimetric assays were carried out [31]. Briefly, Minolta CR-300 colorimeter (Konica Minolta Sensing Inc., Osaka, Japan) with 8 mm diameter aperture, D65 illuminant, 10° standard observer and calibrated according to the manufacturer’s specifications. The CIELAB color scale was applied. L* represents luminosity between 0-black and 100-white; a* distinguishes between green −60 and red +60 and b* between blue -60 and yellow +60. For color measurement, GM (5 g) and meat discs’ samples were placed on plastic plates 5 cm in diameter. Previously, calibration of the equipment was carried out using a white ceramic plate. Three measurements of the chromaticity coordinates (L*, a* and b*) were conducted on the surface of each meat experimental system at different locations per sample over incubation time. In addition, the total color difference ∆E (i.e., magnitude that numerically expresses the color difference perceived by the human eye) between control and inoculated beef without and with LA was calculated for the following comparisons: (i) control GM vs. GM + LAB strains + EHEC, (ii) control GM vs. GM + LAB strains + EHEC + LA, (iii) control meat discs vs. Meat discs + LAB strains + EHEC and (iv) control meat discs vs. meat discs + LAB strains + EHEC + LA. ∆E was calculated according to [31] with the following equation:

$$\Delta E=\sqrt{{\left(L-L_{0}\right)}^{2}+{\left(a-a_{0}\right)}^{2}+{\left(b-b_{0}\right)}^{2}}$$

where L_0_, a_0_, b_0_ are color parameters of the control meat system (un-inoculated and without LA) and L, a, b are color parameters of the inoculated systems without or with LA. ΔE is designated as the total color difference.

### 2.6. Adhesion Assays on Meat Discs

The ability of enterohemorrhagic *Escherichia coli* to attach to meat was analyzed using the method of Marín et al. [32] with modifications. Briefly, the meat discs were aseptically placed inside a flask containing 30 mL of the different cell suspensions (CRL681, CRL35 and/or EHEC) (6 log CFU/mL). It was held for 20 min at room temperature for cell adhesion to the meat surface, then transferred to another flask containing 100 mL of physiological solution and gently shaken 25 times over a period of 15 s to release the loosely attached bacteria. After removing meat discs, the remained solution was used for plating on the selective media. Subsequently, strongly adhered bacterial counts were evaluated by placing the discs in a Stomacher bag together with 100 mL of physiological solution, which was processed in a Stomacher bag for two cycles of two minutes each and plated on selective media. Results were expressed as log CFU/cm^2^.

#### Preparation of Inoculated Meat Discs for Scanning Electron Microscopy (SEM)

After cell adhesion to meat discs, they were gently washed with physiological solution, and a small piece of meat of approximately 1 cm^2^ was cut. The samples were placed in Karnovsky fixative solution (1.7% glutaraldehyde—2.7% paraformaldehyde in phosphate buffer, pH 7.2) for 24 h. Next, dehydration was carried out with successive alcohol strength solutions. First, 30° alcohol was used followed by 50°, 70°, 90° up to absolute alcohol (100°) by 20 min passages. Then, two passages of 30 min with 100% acetone were carried out. The critical drying point was determined with CO_2_ in a Denton Vacuum equipment model DCP-1. The samples were mounted on aluminum stubs, adhered with double-sided carbon conductive adhesive tape, then coated with gold in an Ion Sputter equipment JEOL model JFC-1100. Finally, they were observed in a Zeiss Scanning Electron Microscope model SUPRA 55VP.

### 2.7. Statistical Analysis

Experiments were carried out three times; values and standard errors were calculated from data with three replicates. Statistical analysis of the data was performed with ANOVA and Tukey’s mean comparison tests (*p* ≤ 0.05), while using the InfoStat statistical package to identify significant differences in the parameters analyzed.

## 3. Results

### 3.1. Performance of LAB and Escherichia coli O157:H7 in Meat Experimental Systems 

#### 3.1.1. Ground Meat (GM)

**Figure 2:**
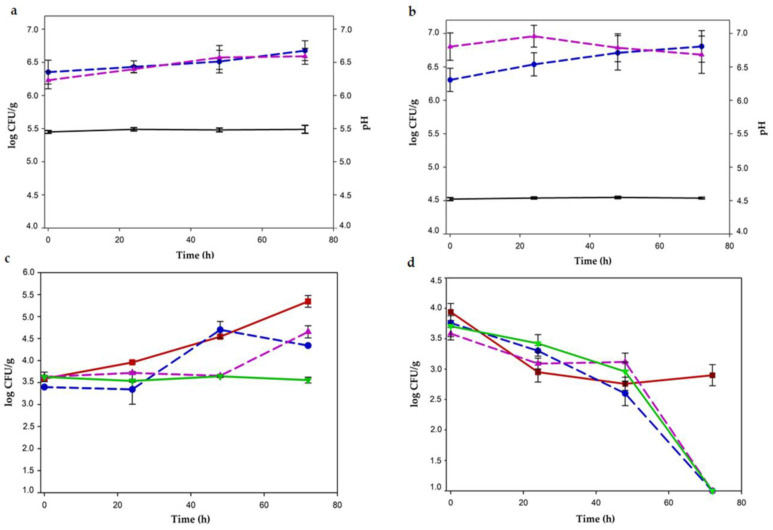
LAB and EHEC growth kinetics (log CFU/g) in individual culture and in co-culture on meat discs at 8 °C. (**a**) pH (black line) and individual growth of *L. plantarum* CRL681 (blue dotted line) and *E. mundtii* CRL 35 (magenta dotted line) without lactic acid (LA). (**b**) pH (black line) and individual growth of CRL681 (blue dotted line) and CRL35 (magenta dotted line) with the addition of 0.6% LA. (**c**) EHEC growth in individual culture (red line) and influenced by the presence of LAB (*L. plantarum* + EHEC, blue dotted line; *E. mundtii* + EHEC, magenta dotted line and *L. plantarum* CRL681 + *E. mundtii* CRL35 + EHEC, green line) without the addition of LA. (**d**) Viability of EHEC in individual culture (red line) and in co-culture in the presence of LAB (*L. plantarum* + EHEC, blue dotted line; *E. mundtii* + EHEC, magenta dotted line and *L. plantarum* CRL681 + *E. mundtii* CRL35 + EHEC, green line) with the addition of 0.6% LA.

When *L. plantarum* CRL681 and *E. mundtii* CRL35 were inoculated individually in GM without LA; low growth of both LAB, with increases between 0.32 and 0.36 log units, respectively, at the end of storage were recorded (Figure 2a). When 0.6% LA was added to the GM, *L. plantarum* CRL681 increased by 0.5 log at 72 h, while *E. mundtii* CRL35 maintained approximately the same numbers during the incubation time (Figure 2b).

Constant pH values all over the storage period (pH 5.5 and 4.5, respectively) in GM without and with LA were found (Figure 2a,b). In the presence of LA, a color change was visually evident after 24 h, turning red ground meat into a brownish color.

On the other hand, growth from 24 h up to 72 h was observed for EHEC inoculated as an individual culture without LA addition (Figure 2c), with an increase by approximately 2 log CFU/g. In addition, when LAB strains, individually or as mixed cultures were co-inoculated with the pathogen, differential growth kinetics were shown for EHEC. In the presence of *L. plantarum* CRL681, EHEC exhibited similar growth of that of the monoculture until 48 h; thereafter, the stationary phase was reached. Whereas, when *E. mundtii* CRL35 was co-inoculated with the pathogen, EHEC counts were almost constant during the first 48 h, followed by a sudden increase up to the end of the storage period. However, when both LAB strains were co-inoculated with the pathogen, *Escherichia coli* did not modify their cell number during the 72 h of storage, showing a bacteriostatic effect (Figure 2c). 

Furthermore, results of EHEC viability when 0.6% lactic acid was added to GM are depicted in Figure 2d. When the pathogen as monoculture was inoculated with lactic acid, a decrease of approximately 1 log CFU/g during the first 24 h was observed; cell concentration remained constant at 3 log CFU/g until the end of incubation. EHEC viability was adversely affected by 0.6% LA, although it did not completely eliminate the pathogen. In contrast, when GM + 0.6% LA + EHEC was inoculated with one or both LAB strains, the viability of *Escherichia coli* decreased gradually until 48 h, becoming undetectable at 72 h. A bactericidal effect occurred from the synergistic action exerted between LAB strains and lactic acid (Figure 2d).

#### 3.1.2. Meat Discs 

**Figure 3:**
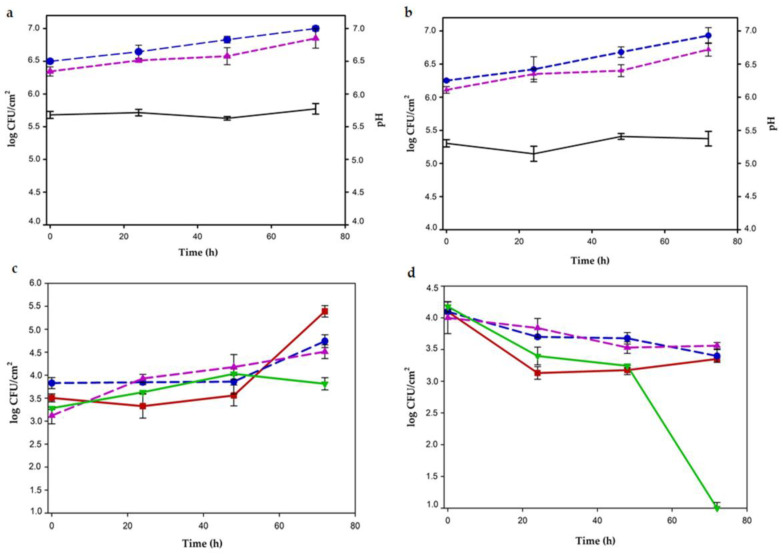
LAB and EHEC growth kinetics (log CFU/cm^2^) in individual culture and in co-culture on meat discs at 8 °C. (**a**) pH (black line) and individual growth of *L. plantarum* CRL681 (blue dotted line) and *E. mundtii* CRL 35 (magenta dotted line) without lactic acid (LA). (**b**) pH (black line) and individual growth of CRL681 (blue dotted line) and CRL35 (magenta dotted line) with the addition of 0.6% LA. (**c**) EHEC growth in individual culture (red line) and influenced by the presence of LAB (*L. plantarum* + EHEC, blue dotted line; *E. mundtii* + EHEC, magenta dotted line and *L. plantarum* CRL681 + *E. mundtii* CRL35 + EHEC, green line) without the addition of LA. (**d**) Viability of EHEC in individual culture (red line) and in co-culture in the presence of LAB (*L. plantarum* + EHEC, blue dotted line; *E. mundtii* + EHEC, magenta dotted line and *L. plantarum* CRL681 + *E. mundtii* CRL35 + EHEC, green line) with the addition of 0.6% LA.

In vacuum-sealed meat discs, the growth of LAB when inoculated as pure cultures increased 0.5 log units during 72 h in both conditions, without and with the addition of 0.6% LA. The pH remained almost constant throughout the incubation period (5.6 and 5.3 without and with LA, respectively) (Figure 3a,b). On the other hand, in the batch where EHEC was inoculated without LA, initial cell counts were maintained for 48 h and then growth was increased more than 1.5 log CFU/cm^2^ up to 72 h (Figure 3c). When each LAB strains was co-inoculated with the pathogen, there was an increase of *Escherichia coli* counts of 1 log CFU/cm^2^ at 72 h. As observed in GM, the greatest antagonistic effect was produced when LAB strains were combined. In this condition, EHEC grew only 0.5 log CFU/cm^2^ as an almost bacteriostatic effect (Figure 3c).

When meat discs were previously treated with 0.6% lactic acid, a significant and differential antagonist effect against EHEC was observed. Inoculated as monoculture, EHEC numbers gradually decreased during the first 24 h, then a constant growth until 72 h was observed (Figure 3d). A bacteriostatic effect was registered when each LAB strain was inoculated independently (Figure 3d). However, an EHEC bactericidal effect was observed when the discs with 0.6% LA were inoculated with both LAB strains. Indeed, EHEC viability progressively decreased up to 48 h, then a dramatic decline of growth occurred to non-detectable counts at 72 h (Figure 3d). The discs treated with 0.6% LA registered a constant pH of 5.3–5.4 throughout the incubation period. 

As a whole, the addition of 0.6% LA to both meat experimental systems produced a significant improvement in the inhibitory action of LAB strains against EHEC. 

### 3.2. Color Evaluation

The instrumental color parameters of experimental meat systems were correlated with the color changes visually observed. L*, a* and b* parameters were statistically analyzed by comparison of each batch at different storage times and different batches at one time point (Table 2 and Table 3).

#### 3.2.1. Ground Meat

When ground meat (GM) was inoculated with LAB and EHEC with or without 0.6% LA, a more rapid and pronounced loss of redness was visually observed over time compared to the control (GM). Indeed, un-inoculated control samples exhibited a significant change in the three color parameters (L*, a* and b*) at 72 h; a predominant brown color was visually evident in GM. On the other hand, batches inoculated with LAB strains + EHEC without the addition of LA showed a significant difference in a* values at 48 h, while batches added with LA presented significant differences in the three parameters (L*, a* and b*) at 48 h of incubation (Table 2).

When the three assayed conditions (control, inoculated and inoculated + LA) were compared, it was found that meat color started to change at 24 h, which was slightly more pronounced when LA was added. Significant changes in L* and a* parameters were observed in the presence of LA compared to those of the control (Table 2). By coincidence, a more rapid loss of reddish color was visually evident under this condition. In addition, no statistical differences in a* and b* parameters of control and inoculated conditions (without and with LA) were shown at 72 h, with all batches exhibiting a brownish color. However, compared to 0 h, a decrease of these parameters was observed in all batches.

#### 3.2.2. Vacuum-Sealed Meat Discs

Colorimetric results obtained for meat discs are shown in Table 3. Non-significant changes in L*, a* and b* parameters were produced neither among batches nor during the storage time. Inoculated conditions (with and without LA) exhibited similar values compared to the control. Therefore, the characteristic meat red color was preserved regardless the presence of bacteria or 0.6% LA.

**Figure 4:**
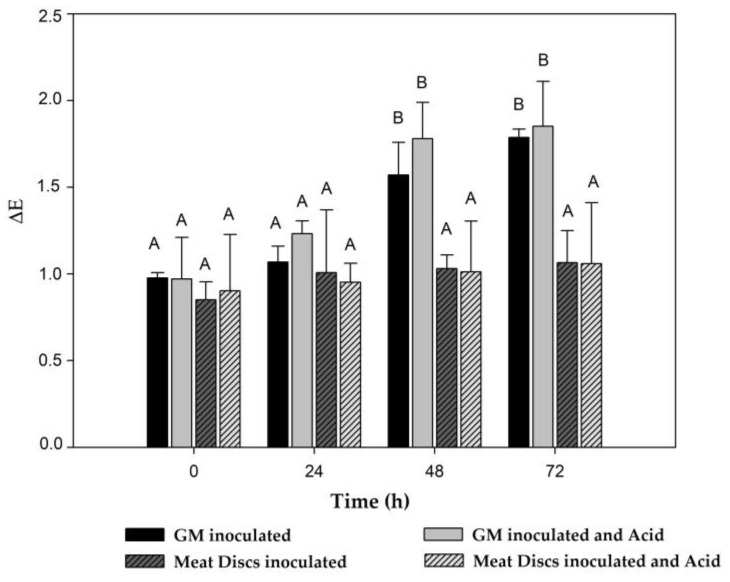
Comparison of total color differences (∆E) in ground meat (GM) and meat disc systems during storage time. Different letters represent significant difference *p* > 0.05. Black bars: GM inoculated (ground meat inoculated with *L. plantarum* CRL681 + *E. mundtii* CRL35 and *Escherichia coli* NCTC12900); grey bars: GM inoculated and acid (ground meat inoculated with the addition of 0.6% lactic acid); gray dashed bars: meat discs inoculated (meat discs inoculated with *L. plantarum* CRL681 + *E. mundtii* CRL35 and *Escherichia coli* NCTC12900); white dashed bars: meat discs inoculated and acid (meat discs inoculated with the addition of 0.6% lactic acid).

In order to better distinguish among different batches, the instrumental color changes during the storage period (i.e., the total color variation, ∆E) between control and inoculated beef without and with lactic acid was determined. Results showed similar ∆E at 0 and 24 h for all batches of both meat experimental systems (Figure 4). However, color variation of GM (all conditions) was significantly different from that of meat discs (all conditions) at 48 and 72 h. Thus, meat discs retained an approximately constant ∆E value throughout the storage period in all evaluated conditions, while GM presented higher ∆E after 24 h in all assayed conditions (Figure 4). 

### 3.3. Adhesion of EHEC and LAB Strains on Meat

**Figure 5:**
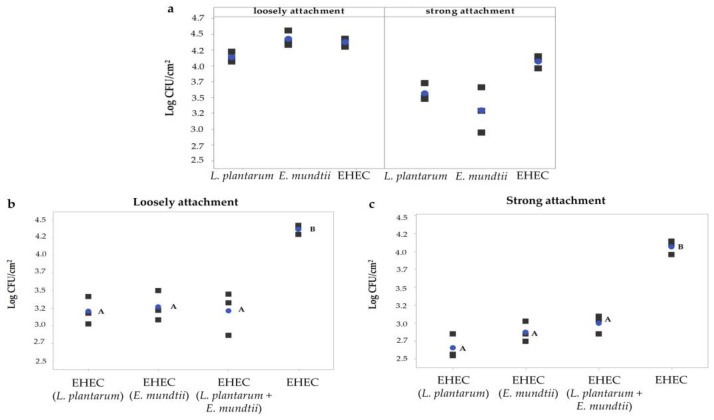
Cells counts of EHEC (*E. coli* O157:H7 NCTC12900) and LAB (*L. plantarum* CRL681; *E. mundtii* CRL35) adhered to meat. (**a**) Strong and loose attachment of single strains on meats discs; (**b**) performance of EHEC in the presence of LAB during loose attachment; (**c**) performance of EHEC in the presence of LAB during strong attachment. The blue dots represent the mean value, and the grey squares represent each independent experiment value. Different letters indicate statistically significant differences between the groups (*p* < 0.05).

When bacterial attachment to meat surface was assayed, results showed that all bacteria (CRL681, CRL35 and *Escherichia coli* NCTC12900) were capable of adhering to meat discs when evaluated individually. Slightly higher counts for loosely than for strongly attached cells were observed (Figure 5a). LAB adhesion was not affected by either the presence of the other LAB strain or the pathogen, retaining its counts similar to those of pure culture. Regarding the adhesion performance of *Escherichia coli* on meat discs, adherence was significantly affected by the presence of LAB strains. In fact, loose and strong attachments decreased by 1.1 ± 0.30 and 1.2 ± 0.17 log CFU/cm^2^, respectively, compared to the adhesion on pure culture (Figure 5b,c). Interestingly, no synergistic action of LAB strain combination on EHEC attachment was observed.

#### Adhesion of Bacterial Strains on Meat Discs by Scanning Electron Microscopy

**Figure 6:**
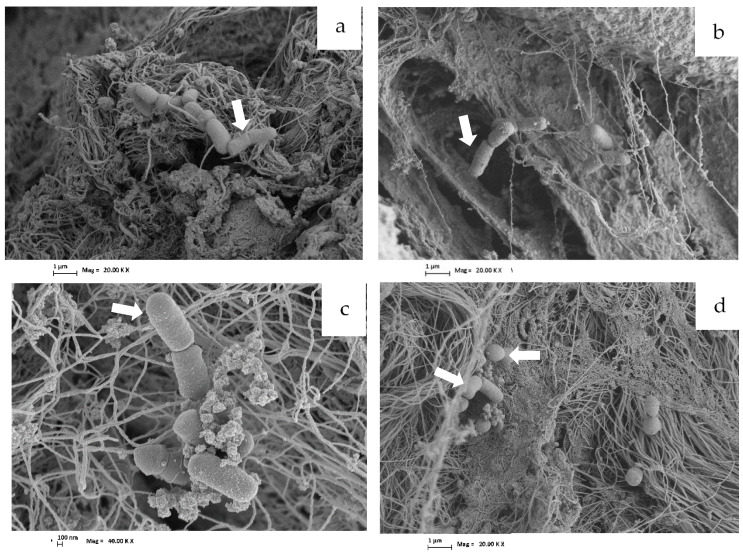
Images obtained from meat discs using scanning electron microscopy. (**a**) EHEC attachment to the meat surface (control); (**b**) EHEC + *L: plantarum* CRL681; (**c**) *L. plantarum* CRL681; (**d**): *E. mundtii* CRL35 and EHEC. Arrows indicate bacilli and cocci bacterial cells. Magnifications: 20 KX (**a**,**b**,**d**) and 40 KX (**c**).

The microphotographs of meat discs after bacterial attachment provide visual information regarding bacterial adhesion to the meat surfaces. They show the skeletal muscle, involving mainly fibers bound together by a framework of extracellular connective tissue along with the inoculated bacteria. Meat inoculated with EHEC (control) is observed in Figure 6a, while *L. plantarum* CRL681 + EHEC is observed in Figure 6b. The three bacteria, CRL681 (bacilli) and CRL35 (cocci) together with EHEC, adhered to meat discs’ surface as shown in Figure 6c,d. It was not possible to differentiate EHEC from *L. plantarum* CRL681 cells because both are bacilli of similar size. Bacterial cells presented optimal shape and integrity, indicating that the adhesion to meat would not be a stressing process for bacterial cells. In addition, low crowded bacterial populations were perceived in SEM images in accordance with the relatively low cell concentrations obtained during the assay of meat attachment (Figure 6).

## 4. Discussion

The use of LAB as bioprotective agents for meat is a promissory and sustainable strategy to preserve meat from spoilage. However, to the best of our knowledge, LAB cultures specifically active against EHEC are not yet available in the market for meat and meat products; the majority of bioprotective LAB are marketed to control *Listeria monocytogenes* (SafePro^®^ B-LC-48, SafePro^®^, ImPorous, Bactoferm^®^, Floracarn). In this study, we evaluated a new strategy involving two LAB strains and low lactic acid concentrations to specifically inactivate this pathogen in meat. In previous studies, the antagonistic ability of the studied strains (*L. plantarum* CRL681 and *E. mundtii* CRL35) against *Escherichia coli* O157:H7 was evident in co-cultures using a meat-based medium at 30 °C. It was also demonstrated that the inhibitory activity of LAB toward EHEC was not due to the production of acid or bacteriocins [2,25]. Herein, the antagonistic potential of these two strains was evaluated under technological conditions using ground meat and whole beef cuts (meat discs) as experimental systems. Results showed that *L. plantarum* CRL681 and *E. mundtii* CRL35 used as mixed cultures produced a bacteriostatic effect on EHEC growth in both meat model systems. However, a bactericidal action of LAB strains was found when meat systems were supplemented with 0.6% of LA, achieving undetectable EHEC counts at 72 h (>4 log reduction). In agreement with our study, reductions of EHEC (2–3 log units) in ground beef after 5 days at 5 °C by using a co-culture of four LAB strains were reported [20,33]. As stated above, undetectable EHEC levels obtained after LAB plus 0.6% LA represents a promissory strategy for a complete pathogen inactivation, which is of great importance when considering the low infective dose of *E. coli* O157:H7 (10 to 100 cells) [34].

The hurdle technology proposed by Leistner (2000) [35] is based on the inhibition of microbial growth by combining the effects of several preservation factors such as temperature (high or low), water activity, acidity (pH), redox potential, chemical additives and/or competitive microorganisms, which if used individually should be applied at extreme levels, generally causing alteration of the characteristics of the food. According to this study, lactic acid (LA) was assayed as an additional hurdle. This acid is commonly used during meat processing as a decontaminating additive and is generally applied in a concentration up to 5% in meat carcasses [36,37,38]. Moreover, considering current consumer demands for minimal interventions in order to preserve as far as possible the original/natural features of foods, the addition of lower lactic acid concentration was used here (from 0.2 to 2% LA). Results showed that 0.6% LA was the minimal concentration that produced a synergistic effect when combined with both LAB strains; this resulted in a significant reduction of the pathogen in both assayed experimental systems. Even though LA use is allowed as a carcass decontaminant, it is known that *Escherichia coli* O157:H7 harbors acid resistance mechanisms, inducing high tolerance to acidic environments [39]. This would explain the low pathogen reductions observed when 0.6% LA was only applied. As a unique pathogen inhibitor, higher LA concentrations (2% to 5%) were reported for moderate reduction achievements (0.8 to 1.4 log CFU/cm^2^) of EHEC in beef [36,40].

On the other hand, even though strategies proposed for pathogen elimination must be efficient, they must also preserve desirable visual and sensory characteristics of the meat products, since they will determine their acceptance by consumers [41]. An important criterion during the selection of bioprotective strains is their ability to stabilize their cell numbers during storage to avoid pH reduction which could compromise the sensory quality of foods. In this study, LAB strains maintained cell viability approximately from the initial numbers (6–7 log CFU/g or cm^2^) all throughout the storage time with constant pH values. In contrast, problems in the acceptability of food products due to LAB growth during storage were reported [19]. In fact, acid production during bacterial growth led to color alterations with high impact on sensorial features. Moreover, when LA at high concentrations is used as meat decontaminant, a loss of reddening is a concern, which is a critical point for its implementation to reduce *Escherichia coli* O157:H7 in meat [42]. Thus, in the present study, a much lower concentration (0.6%) of lactic acid than those commonly used was applied.

The color of red meat represents a fundamental characteristic as it is an indicator of food quality and freshness for consumers. It is known that myoglobin is the pigment responsible for meat’s red color. When meat comes into contact with oxygen, myoglobin is rapidly transformed into oxymyoglobin (MbO), producing the characteristically accepted bright red color; in contrast, myoglobin progressive oxidation form metmyoglobin (MMb) produces a brownish color. The formation of MMb constitutes an undesirable issue, since it determines the shelf life of meat [43]. A loss of red color in ground beef inoculated with LAB + EHEC was observed regardless of the addition of 0.6% LA. However, the reddish color was not affected in the vacuum-sealed discs over the experimentation period and between conditions. In addition, ΔE (total color variation) has been used as a tool to relate with the percentage of MMb in meat; a meat sample with ΔE = 1 indicates 0% MMb while ΔE = 2 corresponds to 20% MMb: the latter was established as a critical point of consumer acceptance [15,41,44]. On this basis, results for meat discs exhibited a stable ΔE = 1 during 72 h, which would not compromise consumer acceptance of bioprotected meat. Conversely, higher values of ΔE (>1.5) after 48 h found for ground meat suggest a limit for acceptance by consumers. The reduction of redness when the GM experimental system was assayed may be related to the higher meat surface exposed to acid when compared with the meat discs’ experimental system; in fact, lower pH values were registered in GM inoculated with LAB bioprotective strains and 0.6% LA.

It is known that meat contamination by EHEC occurs during slaughtering/processing, with bacteria being transferred from the skin to the skeletal muscle. Muscle fibers are surrounded by an extracellular matrix composed mainly by fibrillar collagen, laminin and elastin where bacterial adhesion occurs [45]. A decrease of pathogen adhesion to meat surface (meat discs) when bioprotective LAB were inoculated was demonstrated in the present study. No additive effect on the impaired EHEC adhesion was registered when both LAB strains were combined. When the mix of LAB strains was inoculated with EHEC (1:1), it could act as a unique Gram-positive population competing with Gram-negative pathogenic cells; thus, no additive effect would be produced. Accordingly, *Escherichia coli* O157:H7 NCTC12900 adhesion to collagen IV and laminin was impaired when *E. mundtii* CRL35 was inoculated as a co-culture [25]. It may be suggested that *L. plantarum* CRL681 and *E. mundtii* CRL35 (pure or mixed cultures) act as blocking agents, avoiding the establishment of the pathogen on meat surfaces during meat processing.

## 5. Conclusions

As a result of this study, the use of LAB and 0.6% LA represents a novel effective intervention to inhibit EHEC in meat. Indeed, an undetectable number of EHEC was achieved at 72 h. LAB exerted their antagonistic activity while maintaining minimal growth and a constant pH in meat. EHEC inactivation was produced by both the synergistic effect of LAB strains + 0.6% LA as well as the decreased adhesion of the pathogen to meat by bioprotective LAB. Moreover, the original red color was preserved in vacuum-sealed meat discs during storage. The use of *Lactiplantibacillus plantarum* CRL681 and *Enterococcus mundtii* CRL35 plus 0.6% LA as coadjuvant, represents a new, effective, feasible and ecofriendly strategy to inactivate *Escherichia coli* O157:H7 in meat. Additional technological assays are ongoing to avoid color modifications of ground meat.

## Figures and Tables

**Table 1:** Batches used for the analysis of the antagonistic capacity of LAB with and without 0.6% lactic acid (LA).

Experimental Systems	Ground Meat (GM)	Meat Discs
**Without LA**	1. Control	1. Control
	2. *L. plantarum* (1 × 10^6^ CFU/g)	2. *L. plantarum* (1 × 10^6^ CFU/cm^2^)
	3. *E. mundtii* (1 × 10^6^ CFU/g)	3. *E. mundtii* (1 × 10^6^ CFU/cm^2^)
	4. EHEC (1 × 10^4^ CFU/g)	4. EHEC (1 × 10^4^ CFU/cm^2^)
	5. EHEC + *L. plantarum*	5. EHEC + *L. plantarum*
	6. EHEC + *E. mundtii*	6. EHEC + *E. mundtii*
	7. EHEC + *L. plantarum* + *E. mundtii*	7. EHEC + *L. plantarum* + *E. mundtii*
**With 0.6% LA**	8. Control	8. Control
	9. *L. plantarum* (1 × 10^6^ CFU/g)	9. *L. plantarum* (1 × 10^6^CFU/cm^2^)
	10. *E. mundtii* (1 × 10^6^ CFU/g)	10. *E. mundtii* (1 × 10^6^ CFU/cm^2^)
	11. EHEC (1 × 10^4^ CFU/g)	11. EHEC (1 × 10^4^ CFU/cm^2^)
	12. EHEC + *L. plantarum*	12. EHEC + *L. plantarum*
	13. EHEC + *E. mundtii*	13. EHEC + *E. mundtii*
	14. EHEC + *L. plantarum* + *E. mundtii*	14. EHEC + *L. plantarum* + *E. mundtii*

**Table 2:** CIE Lab parameters in ground meat (GM) at the different conditions assayed during 72 h of incubation at 8 °C.

Condition	Parameter	0 h	24 h	48 h	72 h
Ground meat (GM) control	L*	37.12 ± 0.54 ^a,A^	37.12 ± 0.48 ^a,A^	37.48 ± 0.94 ^a,A^	39.01 ± 0.53 ^b,A^
	a*	15.48 ± 0.22 ^a,A^	15.48 ± 0.32 ^a,A^	15.07 ± 0.40 ^a,A^	14.03± 0.50 ^b,A^
	b*	9.86 ± 0.79 ^a,A^	9.77 ± 0.54 ^a,A^	9.08 ± 0.41 ^ab,A^	8.12 ± 0.75 ^b,A^
GM inoculated with LAB and EHEC	L*	37.36 ± 0.46 ^a,A^	37.57 ± 0.15 ^a,AB^	38.09 ± 0.64 ^a,A^	39.82 ± 0.26 ^b,AB^
	a*	15.66 ± 0.55 ^a,A^	15.79 ± 0.35 ^a,AB^	14.25 ± 0.50 ^b,AB^	12.84 ± 0.66 ^c,A^
	b*	9.26 ± 0.55 ^a,A^	9.54 ± 0.66 ^a,A^	9.10 ± 0.79 ^a,A^	7.64 ± 0.46 ^b,A^
GM inoculated with LAB, EHEC and 0.6% LA	L*	37.09 ± 0.16 ^a,A^	37.95 ± 0.20 ^b,B^	38.01 ± 0.33 ^b,A^	39.94 ± 0.06 ^c,B^
	a*	15.74 ± 0.38 ^a,A^	14.87 ± 0.29 ^a,B^	13.87 ± 0.16 ^b,B^	12.98 ± 0.55 ^b,A^
	b*	9.42 ± 0.19 ^a,A^	9.25 ± 0.16 ^a,A^	8.30 ± 0.43 ^b,A^	7.82 ± 0.62 ^b,A^

Statistical analyses were performed for all conditions at a given time (uppercase letters) and for each one condition during storage time (lowercase letters). For each parameter, means with different letters were statistically different at *p* < 0.05.

**Table 3:** CIE Lab parameters in meat discs at the different assayed conditions during 72 h of incubation at 8 °C.

Condition	Parameter	0 h	24 h	48 h	72 h
Control discs	L*	37.21 ± 0.39 ^a,A^	37.34 ± 0.49 ^a,A^	37.60 ± 0.42 ^a,A^	37.77 ± 0.31 ^a,A^
	a*	15.27 ± 0.81 ^a,A^	15.06 ± 0.24 ^a,A^	15.16 ± 0.36 ^a,A^	15.16 ± 0.66 ^a,A^
	b*	9.79 ± 0.28 ^a,A^	9.80 ± 0.83 ^a,A^	9.72 ± 0.67 ^a,A^	9.51 ± 0.27 ^a,A^
Discs inoculated with LAB and EHEC	L*	37.12 ± 0.19 ^a,A^	37.44 ± 0.31 ^a,A^	37.31 ± 0.31 ^a,A^	37.45 ± 0.72 ^a,A^
	a*	15.05 ± 0.33 ^a,A^	15.35 ± 0.52 ^a,A^	15.17 ± 0.10 ^a,A^	15.25 ± 0.36 ^a,A^
	b*	9.98 ± 0.65 ^a,A^	9.62 ± 0.43 ^a,A^	9.16 ± 0.34 ^a,A^	9.17 ± 0.33 ^a,A^
Inoculated discs and lactic acid	L*	37.30 ± 0.61 ^a,A^	37.48 ± 0.27 ^a,A^	37.17 ± 0.12 ^a,A^	37.29 ± 0.20 ^a,A^
	a*	15.04 ± 0.30 ^a,A^	15.18 ± 0.51 ^a,A^	15.02 ± 0.39 ^a,A^	15.10 ± 0.29 ^a,A^
	b*	9.48 ± 0.59 ^a,A^	9.29 ± 0.53 ^a,A^	9.25 ± 0.15 ^a,A^	9.01 ± 0.37 ^a,A^

Statistical analyses were performed for all conditions at a given time (uppercase letters) and for each one condition during storage time (lowercase letters). For each parameter, means with different letters were statistically different at *p* < 0.05.

## Data Availability

The data used to support the findings of this study can be made available by the corresponding author upon request.
